# Morphological and Ultrastructural Insights into the Goldfish (*Carassius auratus*) Spleen: Immune Organization and Cellular Composition

**DOI:** 10.3390/vetsci12060517

**Published:** 2025-05-25

**Authors:** Doaa M. Mokhtar, Giacomo Zaccone, Manal T. Hussein

**Affiliations:** 1 Department of Cell and Tissues, Faculty of Veterinary Medicine, Assiut University, Assiut 71526, Egypt; manal.hussein@aun.edu.eg; 2 Department of Anatomy and Histology, School of Veterinary Medicine, Badr University in Assiut, Assiut 19952, Egypt; 3 Department of Veterinary Sciences, University of Messina, 98168 Messina, Italy

**Keywords:** teleost immunity, ellipsoids, melanomacrophage centers, dendritic cells, telocytes, epithelial reticular cells, blood–spleen barrier

## Abstract

The spleen plays a critical role in the immune and hematopoietic systems of teleost fish, functioning as a major secondary lymphoid organ. This study provides a detailed morphological and ultrastructural assessment of the spleen in goldfish (*Carassius auratus*), focusing on its immunological organization and cellular diversity. Through light and transmission electron microscopy, we examined red and white pulps, identifying key features such as melanomacrophage centers (MMCs), ellipsoids, and various immune cell types. The red pulp was rich in sinusoidal capillaries and splenic cords, whereas the white pulp housed lymphocytes, dendritic cells, macrophages, and telocytes, all contributing to immune regulation. Notably, ellipsoids were surrounded by reticular and macrophage sheaths, forming a filtration barrier against pathogens. Ultrastructural analysis revealed diverse immune cells with active morphological traits, including macrophages with pseudopodia and pigment granules, dendritic cells with dendrite-like extensions, and epithelial reticular cells involved in forming the blood–spleen barrier. These findings highlight the complex immunological microarchitecture of the goldfish spleen and its functional relevance in teleost immune responses.

## Simple Summary

This study examines the spleen of goldfish using light and electron microscopy. It identifies key immune structures like red and white pulp, ellipsoids, and MMCs. Various immune cells, such as macrophages, lymphocytes, dendritic cells, and telocytes, were observed. Ellipsoids and the blood–spleen barrier play an important role in filtering pathogens. The findings highlight the spleen’s complex role in fish immune defense.

## 1. Introduction

In teleosts, the spleen is an essential organ that plays crucial roles in blood filtration, immunological response, and hematopoiesis [1]. The spleen, kidney, and thymus are essential for both innate and adaptive immunity since teleosts lack bone marrow and lymph nodes [2]. Like the spleens of higher vertebrates, the spleen of fish is a secondary lymphoid organ with a well-developed immunological architecture composed of white pulp, which contains lymphoid tissues necessary for lymphocyte activation and antigen presentation, and red pulp, which breaks down erythrocytes [3]. It is a key site for phagocytosis, immune cell contact, and antigen processing, all of which greatly enhance systemic immunity [4].

Melanomacrophage centers (MMCs), which are found in fish spleens but are absent in mammals, are essential for immune monitoring and waste management because they trap infections, aged cells, and pigments [5,6]. Furthermore, the organ’s immunological complexity is highlighted by the presence of specialized cells, such as macrophages [7], dendritic cells [8], and lymphocytes [9].

The ellipsoid, a specialized capillary structure encircling the terminal branches of the splenic arterioles, is a distinctive feature of the teleost spleen [10]. Ellipsoids constitute a filtration barrier against blood-borne pathogens and act as the first checkpoint for blood filtration and antigen trapping [11].

Goldfish (Carassius auratus), a popular ornamental and experimental species, serves as a great model for immunological studies [12]. There are notable anatomical variations, especially in the makeup of immune cells and the arrangement of vascular components, even though the overall structure of the fish spleen is comparable to that of higher vertebrates [2]. The ultrastructural characteristics of the goldfish spleen are still largely unknown, despite its critical function in fish immunity. The purpose of this work is to use light and transmission electron microscopy to provide a thorough histological and ultrastructural characterization of the spleen of goldfish. This study will advance our knowledge of the immunological roles of the spleen in teleost fish by investigating the cellular makeup of the white and red pulp, the arrangement of the splenic compartments, and the ultrastructural characteristics of important immune cells and ellipsoids.

## 2. Materials and Methods

### 2.1. Sample Collection

The study was authorized by Assiut University’s Ethics Committee in Egypt (ethical number: 06/202/0191). Fourteen healthy mature goldfish (*Carassius auratus*) were used. To ensure optimal health conditions, the goldfish were maintained in the laboratory for a two-week acclimation period prior to the study. During this time, comprehensive bacteriological and parasitological assessments were conducted. For parasitological evaluation, wet-mount preparations of gill filaments were examined under a light microscope to detect the presence of external parasites [13]. No evidence of parasitic infection was found. Additionally, bacterial smears were collected from the intestinal mucosa, gills, and skin of randomly selected fish and stained using the Gram stain technique [14]. Microscopic examination revealed no signs of significant bacterial growth. Healthy fish were then randomly selected from the aquaria, with an average weight of 32.50 ± 3.60 g and a standard length of 9.50 ± 1.1 cm. The selected fish were humanely euthanized using an overdose of MS-222 (3% tricaine) before tissue sampling [15]. After that, the spleen was removed and prepared for histological examination. All procedures adhere to relevant ethical guidelines and regulations.

### 2.2. Semithin Sections and (Transmission Electron Microscopy) TEM Preparations

For 24 h, the spleens were preserved in a 2.5% paraformaldehyde–glutaraldehyde solution [16]. Following fixation, the samples were osmicated with 1% osmium tetroxide in 0.1 M sodium-cacodylate buffer (pH 7.3) and rinsed in 0.1 M phosphate buffer. The samples were embedded in Araldite after being dehydrated and cleared with ethanol and propylene oxide. Using Richert Ultracuts (Leica, Germany), semithin (1 µm thick) sections were stained with toluidine blue. Ultrathin slices (70 nm thick) were cut with an Ultrotom VRV (LKB, Bromma, Germany), stained with uranyl acetate and lead citrate [17], and analyzed under a JEOL 100CX II (JEOL, Tokyo, Japan) transmission electron microscope at Assiut University’s Electron Microscopy Unit.

### 2.3. Tissue Sampling Considerations

While care was taken to randomly select and process spleen samples, some degree of sampling bias may still exist due to the limited number of fish and the spleen’s structural heterogeneity. To reduce this risk, multiple sections from various splenic regions were examined to ensure representative morphological and ultrastructural observations.

## 3. Results

### 3.1. Light Microscopy

**Figure 1:**
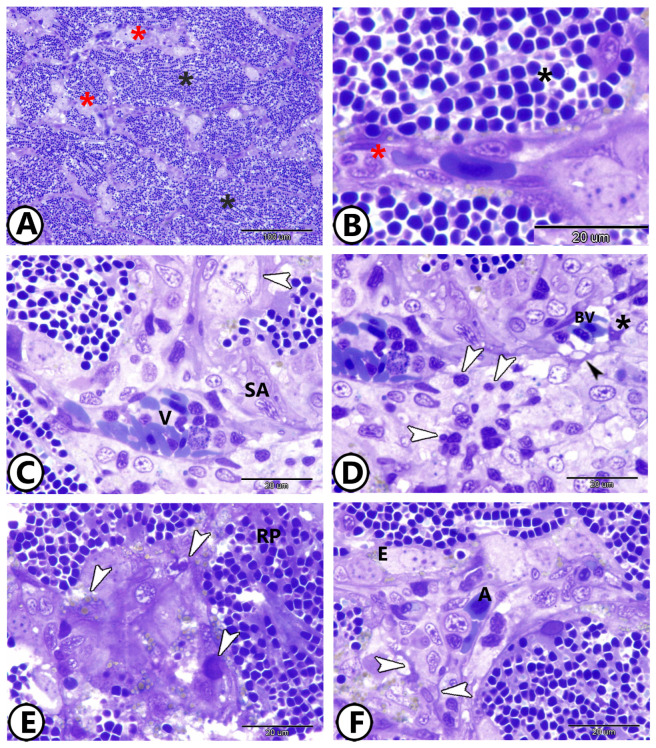
Semithin sections of goldfish’s spleen stained with toluidine blue. (**A**) The spleen consisted of red pulp (black asterisk) and white pulp (red asterisk). (**B**) Sinusoidal capillaries (red asterisk) and splenic cords (black asterisk) constitute the red pulp. (**C**) The division of the splenic arterioles (SAs) produces the ellipsoids (arrowhead). Note the presence of the splenic vein (V)t containing many immune and blood cells. (**D**) The white pulp was made up of leukocyte sheaths, primarily composed of lymphocytes (white arrowheads). Note the presence of telocytes (black arrowhead) around the blood vessel (BV) and their telopodes connected to the dendritic cell (asterisk) and lymphocytes (white arrowheads). (**E**) Melanomacrophage centers (arrowheads) contain pigments in vacuoles. Note the red pulp (RP). (**F**) Splenic cords, which usually encircled ellipsoids (**E**) and arterial vessels (**A**), are a tangle of fibroblast-like cells (arrowheads) with the foci of different blood cells. The scale bar in Figure 1A corresponds to ×40 magnification, while Figure 1B–F were captured at ×100 magnification.

The goldfish’s spleen contained the major splenic components of higher vertebrates, such as ellipsoids, blood arteries, and red and white pulps (Figure 1A). A vast network of sinusoidal capillaries and splenic cords made up the red pulp (Figure 1B). The division of the splenic arterioles produced ellipsoids, which were capillaries with thick walls and a small lumen (Figure 1C). A layer of collagen and reticular fibers encased them. Semithin sections revealed that the white pulp was made up of leukocyte sheaths, primarily composed of lymphocytes (Figure 1D) and macrophages. Telocytes (TCs) were characterized by spindle-shaped cell bodies, and many processes (telopodes) were observed around blood vessels, and their telopodes established heterocellular contacts with dendritic cells and lymphocytes (Figure 1D). Melanomacrophage centers (MMCs) were made up of macrophage aggregates that contained variable quantities of the pigment placed in vacuoles (Figure 1E). There was no germinal center. The majority of the splenic parenchyma was made up by the red pulp (Figure 1E). The splenic cords, which usually encircled ellipsoids and arterial vessels, were foci of different blood cells among a tangle of fibroblast-like cells (Figure 1F).

**Figure 2:**
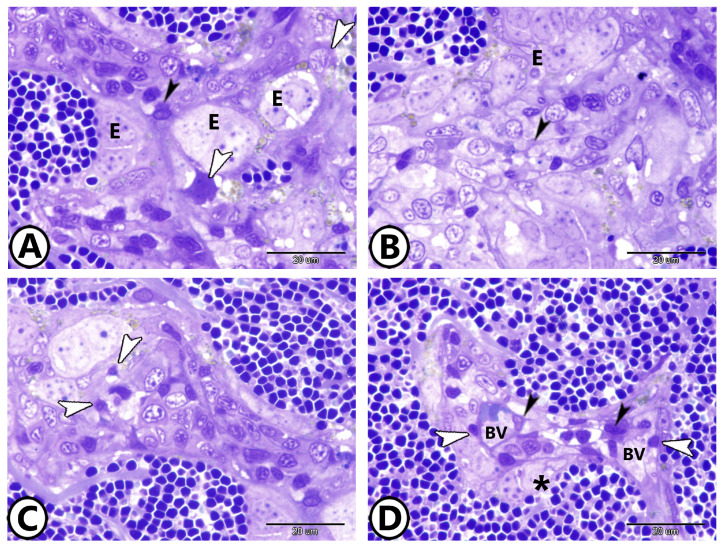
Semithin sections of the spleen of goldfish stained with toluidine blue. (**A**,**B**) The ellipsoids (E) pass through macrophages (white arrowheads) and the sheath of reticular cells (black arrowhead). (**C**) Among the splenic cells, dendritic cells are visible (arrowheads). (**D**) The splenic blood barrier consisted of branching the epithelial reticular cells (black arrowheads) and endothelium (white arrowheads) of blood vessels (BVs) neighboring to the ellipsoids (asterisk).

The cuboidal endothelial cells made up the ellipsoids. They pass through macrophages and the sheaths of reticular cells (Figure 2A,B). Among the splenic cells, dendritic cells with distinctive thin dendrite-like processes were observed (Figure 2C). The splenic blood barrier consisted of branching epithelial reticular cells that extended around the endothelium of blood vessels adjacent to the ellipsoids (Figure 2D).

### 3.2. Electron Microscopy

**Figure 3:**
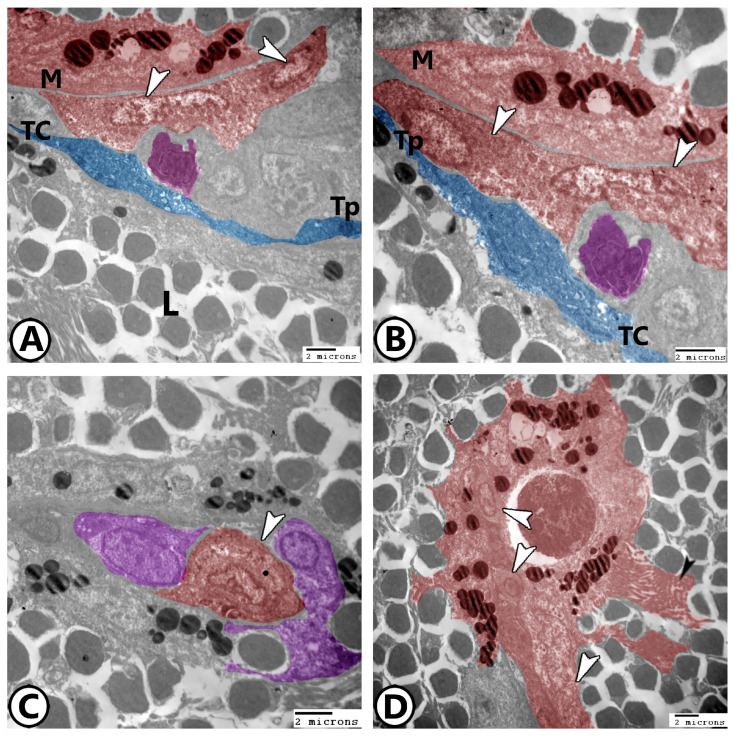
Digital colored TEM image showing (**A**,**B**) Low and higher magnification showing macrophages (red, arrowhead, M), dendritic cells (pink), and telocytes (TC, blue) and their telopodes (Tp). Note the presence of lymphocytes (L). (**C**) Macrophages (red, arrowhead) and branched epithelial reticular cells (violet). (**D**) MMCs (red) consisted of many macrophages (white arrowheads). Note the presence of microvilli (black arrowhead).

The following were the primary cells found in goldfish spleens:

Lymphocytes: One of the most common cell types in the spleen was lymphocytes. They had heterochromatic nuclei and a greater nuclear to cytoplasmic ratio (Figure 3A).

Dendritic cells: They had pseudopodia, and indented heterochromatic nuclei (Figure 3A,B).

Macrophages: The most prevalent cells in the spleen were macrophages. Their cytoplasm contained numerous lysosomes and other phagocytosed materials, and the plasma membrane displayed lengthy pseudopodia. Dendritic cells are typically linked to them (Figure 3A,B). In the splenic white pulp, telocytes with their long telopodes can be investigated in close proximity to dendritic cells and macrophages (Figure 3A,B). The kidney-shaped eccentric euchromatic nucleus (Figure 3C) was a characteristic of irregularly shaped active macrophages. With vesicles containing pigments, they were combined to form melanomacrophage centers (MMCs) (Figure 3D). Phagocytic cells like macrophages may also have surface projections, including microvilli, to facilitate phagocytosis and immune interactions (Figure 3D).

**Figure 4:**
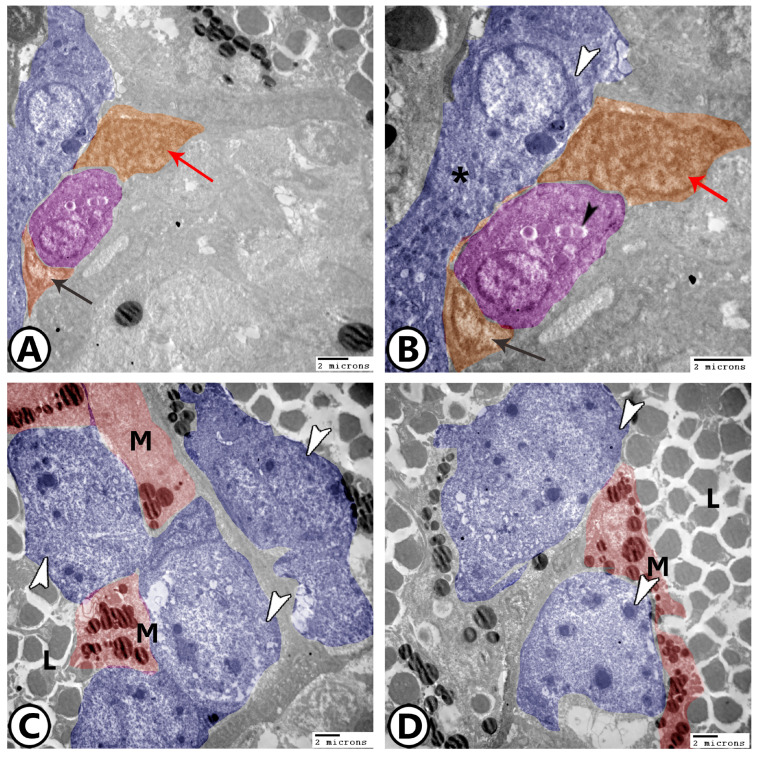
Digital colored TEM image showing (**A**,**B**) Low and higher magnification showing ellipsoids (pink, black arrowhead) and epithelial reticular cells (blue) containing rER (white arrowhead) and mitochondria (asterisk). Note the epithelial reticular cells in association with the flattened (black arrow, orange) and cuboidal endothelium (orange, red arrow) that form the splenic blood barrier. (**C**,**D**) Epithelial reticular cells (blue, arrowheads) and macrophages (red, M). Note the presence of lymphocytes (L).

Ellipsoids: The simple cuboidal epithelium lining the ellipsoids had heterochromatin aggregates in the center of an uneven nucleus. Lysosomes, mitochondria, and vacuoles were seen in the cytoplasm (Figure 4A,B). The epithelial reticular cells in association with the flattened and cuboidal endothelium form the splenic blood barrier (Figure 4A,B). The epithelial reticular cells were branching, with processes extending between the lymphocytes. Their cytoplasm contained many ribosomes, mitochondria, rER, and electron-lucent vesicles, and they have a relationship with macrophages (Figure 4C,D).

**Figure 5:**
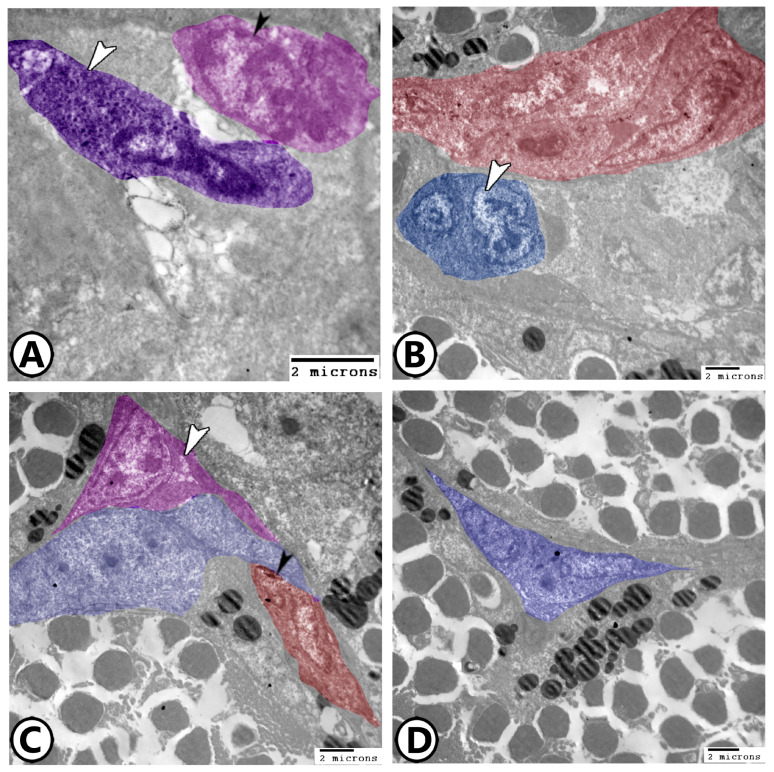
Digital colored TEM image showing (**A**) Neutrophils (violet, white arrowhead) and dendritic cells (pink, black arrowhead). (**B**) Macrophages (red) in the vicinity of mitotic cells (blue, arrowhead). (**C**) Monocytes (red), dendritic cells (pink), and epithelial reticular cells (blue). (**D**) Epithelial reticular cells (blue).

Goldfish spleens were commonly found to include neutrophils (Figure 5A), which were distinguished by their segmented nucleus and densely distributed nuclear chromatin. Numerous electron-dense granules, some phagocytosed items, and vacuoles were seen in the cytoplasm. Some cells with mitotic divisions were observed in the vicinity to macrophages (Figure 5B). Dendritic cells with a high nuclear-to-cytoplasmic ratio, vacuolated cytoplasm, and small processes were detected near epithelial reticular cells. In addition, monocytes with a characteristically large kidney-shaped nucleus could be identified in the splenic white pulp (Figure 5C,D).

## 4. Discussion

Teleosts’ immune systems consist of pronephros, spleen, thymus, and mucosa-associated lymphoid tissues (MALTs) [18,19]. The spleen is the primary secondary lymphoid organ that is essential for antigen trapping [20]. It involves the production of adaptive immunological responses, as well as an enormous number of (IgM + B) lymphocytes [21]. The goldfish’s spleen showed the fundamental components of higher vertebrates, including ellipsoids arising from the division of splenic arterioles, red, and white pulps. In goldfish, no boundaries were observed between the white and red pulps of the splenic parenchyma. The majority of the spleen in teleosts is made up of red pulp. The white pulp is distinguished by lymphocytes that are sporadically dispersed throughout its reticular network and associated with ellipsoids [22].

In chickens, the splenic ellipsoid is a distinctive structure that functions as a filtration and phagocytic barrier [23], soft-shelled turtles (*Pelodiseus sinensis*) [24], and darkbarbel catfish (*Pelteobagrus vachelli*) [25]. According to the current study, ellipsoids are terminal branches of the splenic arterioles that have a thick wall and limited lumen and are encased in a layer of reticular cells and macrophages. These characteristics align with the descriptions of ellipsoids in sunfish [26]. Nonetheless, the lack of these ellipsoids distinguishes other fish species, such as Anguilliformes [27]. Fish splenic ellipsoids have a unique capacity to capture a variety of substances, such as degenerated erythrocytes and infections including *Aeromomas salmonicida* [28], and intravascularly injected materials [29]. It was demonstrated by Furukawa et al. [30] that the ellipsoids filter materials based on their size.

Melanomacrophage centers (MMCs), which are composed of macrophage aggregations with variable pigment levels and located in vacuoles, are clearly visible in the goldfish’s spleen. Usually, these macrophages are related to leukocytes and dendritic cells [31]. In teleosts, the MM is a distinctive and prevalent type of immune cell found in the spleen [32]. Different levels of pigment, such as lipofuscin (yellow to golden brown), hemosiderin, ceroid, or melanin (black to brown), are found in the vacuoles within these cells [33]. MMCs are crucial components of the fish immune system, and they perform phagocytosis, antigen processing, endogenous and exogenous material destruction, detoxification, and recycling [32,34,35]. Additionally, splenic MMCs are a possible biomonitoring instrument for assessing the effects of trace amounts of pesticide pollutants [5,36]. As fish become older, their MMC size and quantity dramatically increase, serving as an indicator of immunological activity [6]. Environmental deterioration and fish health have an impact on the quantity, size, and pigment content of MMCs [37]. It has been proposed that stress and environmental variables, rather than tissue catabolism, are the causes of increased MMCs in the fish spleen [38]. The defense against harmful free radicals generated during unsaturated lipid peroxidation in fish is greatly enhanced by melanin buildup in the macrophages [39]. Melanin synthesis in the spleen is indicated by the expression of genes related to the melanogenesis pathway [40].

An essential part of the splenic immune system is the biological barrier known as the blood–spleen barrier (BSB). Weiss initially discovered it in 1986 in mice [41], and it was discovered to play a role in stabilizing the white pulp microenvironment and providing the white pulp with antigen information [42]. The BSB is located in the marginal zone of mammals [43] and in the peri-ellipsoidal lymphatic sheaths (PELSs) and ellipsoids of species like chickens [23], turtles [24], and Nile tilapia [25], that do not have marginal zones and its presence is indicative of a selective filtration mechanism that likely regulates antigen entry into immune niches.

Among the splenic cells, dendritic cells were identified. These cells are antigen-presenting cells that possess motility, dendritic shape, phagocytic ability, and T cell-catalyzed characteristics [8]. It has been established that several teleosts’ organs, including the spleen, contain functional dendritic cells [3]. These cells are crucial because they provide the essential connections that blood vessel endothelial and lymphoid cells need to function [44]. The detection of neutrophils adds further complexity to the spleen’s immune profile, suggesting a coordinated innate immune response to systemic insults [45]. The presence of mitotic cells near immune populations also increases the possibility of localized hematopoietic activity, which may compensate for the absence of bone marrow in teleosts. Collectively, these features affirm the goldfish spleen’s multifaceted role in both innate and adaptive immunity.

Additionally, our findings underscore the importance of telocytes (TCs) as emerging players in teleost spleen immunology. These interstitial cells, distinguished by their long telopodes, appear to form dynamic cellular networks that interface with lymphocytes, dendritic cells, and macrophages, indicating a role in immune regulation and stromal signaling. This suggests that telocytes may act as sentinels, relaying immune or inflammatory cues through their extended cytoplasmic projections. Their ultrastructural features, including a rich presence of mitochondria and endoplasmic reticulum, further imply metabolic activity consistent with intercellular communication and homeostatic maintenance [46,47]. Recent studies also propose the potential involvement of TCs in guiding immune cell migration and influencing the differentiation of myeloid lineages within the spleen microenvironment [48]. According to a recent study by Huang et al. [49], TCs may play a role in phagocytosis, apoptosis, macrophage development, and the mitochondrial pathway. Furthermore, these findings imply that TCs may be involved in the onset of inflammation.

The structural features of the goldfish spleen represent adaptive responses to the challenges of aquatic environments. Specialized components like ellipsoids and melanomacrophage centers enhance the filtration of bloodborne pathogens and immune surveillance; they have critical functions in pathogen-rich waters [50]. The arrangement of immune cells, including macrophages, dendritic cells, and telocytes, reflects an evolutionary strategy to support efficient antigen processing and immune regulation in the absence of lymph nodes or bone marrow, underscoring the spleen’s central role in fish immunity [44].

Although this study provides valuable insights into the goldfish spleen, potential variations related to sex, size, and age were not specifically addressed. These factors can influence immune organ structure and cellular composition. For instance, the hormonal differences between sexes and developmental changes associated with growth or aging may affect the distribution and activity of immune cells, including melanomacrophage centers and lymphocyte populations [51]. Future studies incorporating sex differentiation, broader size ranges, and age groups would help clarify the extent of such biological variability and strengthen the generalizability of the findings.

## 5. Conclusions

The present study offers a comprehensive histological and ultrastructural evaluation of the goldfish spleen, shedding light on its intricate immune architecture and cellular complexity. The identification of distinct structural components such as ellipsoids, melanomacrophage centers (MMCs), and a variety of immune cells including macrophages, dendritic cells, lymphocytes, and telocytes, emphasizes the spleen’s vital role in teleost immunity. The organization of the red and white pulps, along with the presence of a specialized blood–spleen barrier formed by epithelial reticular cells and endothelial elements, reflects a sophisticated mechanism for the immune surveillance and filtration of bloodborne pathogens. These findings not only contribute to our understanding of the immunological functions of the spleen in the teleost, but also support the use of goldfish as a valuable model for studying comparative vertebrate immunology.

## Data Availability

The article contains the data that were presented in this investigation.
